# Could admission level of uric acid predict total diuretic dose in acute heart failure?

**DOI:** 10.1186/s12872-023-03687-w

**Published:** 2024-01-03

**Authors:** Maryam Chenaghlou, Fatemeh Abedi mahzoon, Sina Hamzehzadeh, Ali Norouzi, Hadi Sahrai, Nasibeh Mohammadi, Negin Khadem haghighi, Mirsaeed Abdollahi, Mohammadreza Taban Sadeghi, Erfan Banisefid

**Affiliations:** 1 Cardiovascular research center, Tabriz University of Medical Sciences, Tabriz, Iran; 2 Student research committee, Tabriz University of Medical Sciences, Tabriz, 5166614756 Iran; 3 Faculty of medicine, Zanjan University of Medical Sciences, Zanjan, Iran

**Keywords:** Heart Failure, Hyperuricemia, Diuretic dosage

## Abstract

**Background:**

Recent studies have shown that increases in serum UA levels are associated with adverse clinical outcomes in patients with chronic heart failure (CHF); the aim of this study was to determine the relationship between serum uric acid and total diuretic dose received during hospitalization in hospitalized patients with acute exacerbation of heart failure. The main purpose of this study is to determine the role of uric acid as a biomarker that can be a substitute for pro-BNP in clinical evaluation and the need for diuretics in hospitalized patients with acute heart failure.

**Methods:**

After approving the plan in the Research Council of the Heart Department and obtaining an ethical code from the Regional Committee on Research Ethics (Human Subjects Studies), the researcher referred to the archives of our center, the case of 100 patients diagnosed with acute heart failure. Cardiac patients were selected, and the information required for the study was collected using a pre-prepared data collection form, and the information was entered into SPSS software after categorization and appropriate analysis and statistical tests were performed on it. Were performed and in all statistical tests the statistical significance level was considered 0.05:

**Results:**

100 patients with acute heart failure were included in this study with a mean age of 63.43 ± 14.78 years. 66% of them were men. The mean dose of furosemide in these patients was 680.92 ± 377.47 mg and the mean serum uric acid level in these patients was 8.55 ± 2.50 mg / dL. In the study of the relationship between the variables, there was a significant relationship between the dose of furosemide received with the serum level of serum uric acid (*P* = 0.017, r = 0.248 and *P* = 0.009, r = -0.267, respectively). There is also a significant relationship between serum uric acid level and patient mortality (*P* = 0.013, r = 0.247). However this relationship lost its significance after multivariate analysis.

**Conclusion:**

There is a significant relationship between serum uric acid level and diuretic use. However, in-hospital mortality is not related to uric acid levels at admission.

**Supplementary Information:**

The online version contains supplementary material available at 10.1186/s12872-023-03687-w.

## Introduction

Heart failure (HF) is a clinical syndrome resulting from injury and congestion of heart with a considerable rate of morbidity and mortality [1]. The prevalence of HF can be estimated at 1–2% in the western countries and the incidence approaches 5–10 per 1000 persons per year. Estimates of the occurrence of HF in the developing countries are largely absent [2]. While the pathophysiology of HF is likely multifactorial, an imbalance in the neuroendocrine systems regulating cardiovascular homeostasis plays a central role in HF. B-Type Natriuretic Peptide (BNP) and N-terminal prohormone of brain natriuretic peptide (NT-proBNP) are synthesized from a pre-hormone of 134 amino acids, encoded by the NPPB gene. Circulating BNP and NT-proBNP levels are normally very low, but increase significantly in HF patients as a mechanism to restore normal hemodynamics. BNP promotes arterial vasodilation, dieresis, and natriuresis, exerts anti-hypertrophic and anti-fibrotic effects, and counteracts the activation of the renin–angiotensin–aldosterone system (RAAS), sympathetic nervous system (SNS) and the endothelin systems [3]. Recent studies suggest the use of BNP and NT-proBNP to diagnose HF [4, 5]. Studies also suggest that high entry BNP levels are significantly associated with in hospital mortality of HF patients [6] and Similarly, NT-proBNP can also predict the short- and long-term prognosis in patients with acute HF [7, 8]. Medical Treatment of HF often focuses on a combination of afterload-reduction with angiotensin-converting-enzyme (ACE) inhibitors, reduction of catecholamine surges with beta blockers, and preload-reduction with diuretics [9, 10]. Diuretics are drugs that increase the flow of urine by acting on the kidneys. Diuretics like furosemide are essential for relieving dyspnea and signs of sodium and water retention (peripheral edema or pleural effusion) [11]. Uric acid (UA) is the end product of purine metabolism by xanthine oxidase (XO). It is produced in the liver and secreted by proximal tubules in kidney. Serum level of UA is the main risk factor for many diseases related to lifestyle in adults, Such as hypertension, diabetes and metabolic syndrome. which in terms of etiology are also related to atherosclerosis [12, 13]. Hyperuricemia is defined as Serum UA level higher than 7 dl/mg in men and higher than 6 dl/mg in women. Hyperuricemia is a common condition in nearly half of patients with HF [14]. In a study by Mantovani et al. patients with HF were classified in terms of serum levels of UA, and patients with higher serum UA levels, had lesser long term survival rate and had a higher risk of hospitalization [15]. Also, in the study of Tamariz et al., it was reported that the Serum level of UA has a linear correlation with the adverse clinical outcome of patients with HF and high serum UA levels (higher than 7 mg/dl) is an independent predictive factor for the mortality of HF patients [16]. In fact, the summary of recent studies shows that Serum UA level is a predictive marker for HF major scoring systems such as Seattle Heart Failure Model and SENIORS mortality risk model [17, 18]. In a study by Misra et al. on 11,681 male patients diagnosed with HF, it was determined that the increase or discontinuation of diuretics can be significantly related to serum level of UA and hyperuricemia [19]. Another research suggests that Prevalence of hyperuricemia in HF patients were 60% and had a significant relationship with diuretic use and serum Brain natriuretic peptide (BNP) levels [20]. Also In the study of Yao et al. on 956 patients with chronic HF who were treated with loop diuretics, It has been determined that the consumption of these diuretics is associated with the average serum level of UA and mortality in these patients [21]. To our knowledge, no study has been done in this regard. In addition, so far, the relationship between the dose of diuretic and the serum level of UA is completely unknown. Considering this, and as UA can be estimated in an easier and cheaper way compared to BNPs, the purpose of the present study is to determine the relationship between Serum UA levels with the total dose of diuretics received during hospitalization in patients hospitalized with acute exacerbations of HF.

## Methods and materials

### Study design and setting

This is a longitudinal analytic study that examined the documents of patients with acute HF as new patients or decompensated chronic HF recorded in the files with convenient sampling from June 2021 to March 2022 in Shahid Madani Hospital in Tabriz, Iran. According to a study by Zhou et al. [22]. and consideration of 1.1 variances in serum UA level, by using Power and Sample Size software (with 90% power and 5% of type 1 error, or alpha), the study sample size was calculated to be 76 patients. To increase the accuracy of the study, 100 patients were included in it. Patients with a history of renal stones, gout, malignancy, significant liver disease, hematologic dyscrasias, or previous acute heart accidents such as myocardial infarction (within one month) were excluded from the study. Furthermore, patients who received anti-hyperuricemia medications such as allopurinol and patients with an eGFR lower than 30 ml/min were excluded from the study.

### Data collection and study performing

We used the most recent guidelines to diagnose HF. All patients were divided into two groups. We measured serum UA levels in all patients at the start of their hospitalization and before the start of diuretic therapy of our patients. Group A comprised 79 patients with hyperuricemia (serum UA levels of more than 7 mg/dl in men and more than 6 mg/dl in women), and group B comprised 21 patients with a normal serum UA level. All demographic information (age, gender, underlying disease, drug history, familial history, smoking status, and alcohol consumption), echocardiographic or electrocardiographic (ECG) findings, and in-hospital outcomes were documented. The diuretic dose received during hospitalization was extracted from the patient’s clinical records and recorded as a cumulative dose during hospitalization. To evaluate kidney function, the serum creatinine level of patients was recorded at the beginning of hospitalization and on the day of discharge (or at most 7 days after discharge), and the estimated GFR (eGFR) of the patients was calculated. Finally, the association between serum UA level and kidney function, in-hospital or short-term outcomes (the need for mechanical ventilation, inotrope, liver failure, worsening of renal function with > 25% or > 0.3 mg/dl increase in creatinine compared to the initial level, the need for dialysis, in-hospital mortality, or discharge with the good general condition), and the cumulative dose of diuretic received was investigated. The amount of volume overload was based on the physicians judgment with consideration of the clinical symptoms such as dyspnea, orthopnea, pulmonary rales, and lower limb edema at the beginning of hospitalization and the resolution of these symptoms and signs during discharge.

### Statistical analysis

All data were analyzed using IBM SPSS 26, and the Kolmogorov-Smirnov test was used to determine the normality of the collected data. According to the distribution of quantitative data, the mean and standard deviation were reported. We also reported the nominal and ordinal variables with frequencies and percentages. The Mann-Whitney U and t-student tests were used to compare differences in variables between the two groups based on various patient characteristics. Correlations between serum UA levels and lengths of admission with other variables were determined by the Spearman correlation coefficient. Univariable and multivariable logistic regression analyses were also employed to investigate the relationship between the variables and short-term (in-hospital) mortality.

### Ethical considerations

The current study was approved by the ethical committee of Tabriz University of Medical Sciences with code IR.TBZMED.REC.1400.513. all methods were carried out in accordance with relevant guidelines and regulations. all experimental protocols were checked by cardiovascular research center then approved by scientific committee of medicine faculty. All patients’ information was kept confidential, and their personal information was not mentioned or published anywhere. Furthermore, all needed tests were performed for patients and no extra fees were charged to the patients. before collecting data, informed consent was obtained from all patients.

## Results

In (Table 1) Demographic information of the patients in this study is shown. 66% of patients were male, and the average age was 63.43 ± 14.78 years. The mean weight in hyperuricemia Group was 74.5 ± 12.75 kg and 58.95 ± 15.76 in the group with normal UA, indicating that the weight of patients with hyperuricemia was significantly higher (*P*-value = 0.026). however, there was no significant association between UA and high body mass index (BMI) based on gender subgroups (*P*-value = 0.07 in female and *P*-value = 0.25 in male). In 43% of cases, HF was caused by ischemic heart problems. However, arrhythmia, renal failure, anemia, and failure to comply with the medication regimen were the other less common causes of decompensated HF. (Table 2) 88% of patients had dyspnea, 24% had orthopnea, and 5% had paroxysmal nocturnal dyspnea (PND). Most patients were in Class III of the NYHA functional classification. The edema in patients with hyperuricemia was significantly higher (*P*-value = 0.028). (Table 3) Beta-blocker medication usage was considerably higher in patients with hyperuricemia (*P*-value = 0.006). The most common medicines given to patients in the hospital were beta-blockers (85%) and Spironolactone (75%). During hospitalization, 27% received an inotrope (mostly milrinone and norepinephrine), and 9% received nitrate-based vasodilators such as isosorbide or nitroglycerin. Furthermore, digoxin was significantly administered to hyperuricemia patients compared to the normal UA group (*P*-value = 0.012). (Table 4) In general, the average serum UA level in patients was 8.55 ± 2.50 mg/dL, which was 8.55 ± 2.50 in the hyperuricemia group and 5.22 ± 1.31 in patients with a normal serum UA level. There were significant differences between the two groups in admission and discharged creatinine levels, which were significantly higher in patients with hyperuricemia (*P*-value = 0.002 and *P*-value = 0.001, respectively). The discharged eGFR level was significantly lower in patients with hyperuricemia (*P*-value = 0.033), and although the admission eGFR level was lower in the hyperuricemia group, it was not statistically significant. (*P*-value = 0.082). Furthermore, the blood sugar level was considerably higher in the hyperuricemia group (*P*-value = 0.022). Other laboratory findings such as complete blood count (CBC), electrolytes, lipid profile, and liver enzymes are described in detail in (Table 5). The mean dose of furosemide received was reported to be 609.95 ± 380.69 mg; The need for intravenous (IV) furosemide differed significantly between the two groups, and it was higher in patients with hyperuricemia (*P*-value = 0.045), although there was no statistically significant difference between the two groups in receiving other diuretics. (Table 6). Based on gender subgroup analysis high serum UA levels had no association with the need for a higher dosage of diuretic therapy in women (Table 7). (Table 8 and 9) show the electrocardiographic and echocardiographic findings in these patients. there were significant positive correlations between some echocardiographic findings such as left ventricular end diastolic diameter (LVED) (*P*-value = 0.014/r = 0.279), left ventricular end-systolic diameter (LVES) (*P*-value = 0.002/r = 0.578), Left Atrial Volume Index (LAVI) (*P*-value = 0.048/r = 0.458), RAA (*P*-value = 0.04/r = 0.474), and Right ventricular dimension at end- diastole (RVDD) (*P*-value = 0.007/r = 0.325) with the serum UA level. However, there was a significantly negative correlation between Left ventricular ejection fraction (LVEF) and the serum UA level, which means a lower ejection fraction is accompanied by a higher serum UA level (*P*-value = 0.014/r = -0.265). Furthermore, we found that higher body weight is significantly correlated with a higher serum UA level (*P*-value = 0.03/r = 0.3). Additionally, there was a significant positive correlation between the total and intravenous furosemide dosage during hospitalization and the serum UA level (*P*-value = 0.005/r = 0.291 and *P*-value = 0.002/r = 0.313, respectively). Also, there was a significant positive correlation between the furosemide dosage before hospitalization and the serum UA level. (*P*-value = 0.008/r = 0.282). Also, higher serum creatinine levels and BUN were significantly correlated with higher serum UA levels (*P*-value = 0.001/r = 0.323 and *P*-value < 0.001/r = 0.371, respectively). Furthermore, there was a significant positive correlation between total, oral, and intravenous furosemide dosage and admission days (all *P*-values < 0.001). Also, we found lower LVED and right-sided aortic arch (RAA) are significantly accompanied by more admission days (*P*-value = 0.01/r = -0.295 and *P*-value = 0.014/r = -0.554, respectively). (Table 10). However these findings lost their significance after multivariate analysis (Table 11). There is a significant relationship between the serum UA level and the mortality (short-term outcome) of the patients (*P*-value = 0.013, r = 0.247). According to univariate logistic regression shown in (Table 12), there was a significant association between the admission UA level and the mortality rate. Also, there was a significant association between the amount of intravenous and total furosemide received with the mortality rate. However, according to multivariate logistic regression shown in (Table 13), there was no independent relationship between any of the examined variables and the in-hospital mortality rate.

**Table 1:** Demographic information

Variables	Unit/Type	hyperuricemia Group (Mean ± SD) (Frequency/Percentage)	normal uric acid Group (Mean ± SD) (Frequency/Percentage)	Total (Mean ± SD) (Frequency/Percentage)	*P*-value
*Gender*	Female	26/32.9	8/38.1	34/34	0.656
	Male	53/67.1	13/61.9	66/66	
*Age*	years	64.62 ± 14.37	58.95 ± 15.76	63.43 ± 14.78	0.154
*Height*	cm	164.13 ± 7.16	167.84 ± 8.49	167.27 ± 8.35	0.243
*Weight*	kg	74.5 ± 12.75	63.88 ± 17.12	72.87 ± 13.87	**0.026**
*Male BMI*	Normal	10/33.3	3/60.0	13/37.1	0.253
	High	20/66.7	2/40.0	22/62.9	0.253
*Female BMI*	Normal	6/42.9	3/100.0	9/52.9	0.072
	High	8/57.1	0/0	8/47.1	0.072
*HTN*	-	47/59.5	12/57.1	59/59	0.846
*Diabetes*	-	32/40.5	7/33.3	39/39	0.549
*Dyslipidemia*	-	9/11.4	2/9.5	11/11	0.808
*CAD*	-	34/43	5/23.8	39/39	0.108
*HF*	-	62/78.5	15/71.4	77/77	0.495
*CKD*	-	16/20.3	1/4.8	17/17	0.093
*Smoking status*	-	28/35.4	9/42.9	37/37	0.532
*Alcohol consuming*	-	2/2.5	0/0	2/2	0.461
*Drug abuse*	-	1/1.3	4/19	5/5	**0.001**
*Employment status*	Employed	39/49.4	9/42.9	48/48	0.178
	unemployed	40/50.6	12/57.1	52/52	

**Table 2:** Causes of decompensated heart failure

Variables	hyperuricemia Group (Frequency/Percentage)	normal uric acid Group (Frequency/Percentage)	Total (Frequency/Percentage)	*P*-value
*Failure to use medications*	0/0	1/4.8	1/1	0.336
*ACS*	26/32.9	6/28.6	32/32	
*Anemia*	1/1.3	1/4.8	2/2	
*Renal failure*	7/8.9	1/4.8	8/8	
*Arrhythmia*	20/25.3	4/19	24/24	
*Unknown causes*	25/31.6	8/38.1	33/33	

**Table 3:** Clinical status of patients

Variables	Unit/Type	hyperuricemia Group (Mean ± SD) (Frequency/Percentage)	normal uric acid Group (Mean ± SD) (Frequency/Percentage)	Total (Mean ± SD) (Frequency/Percentage)	*P*-value
*Blood pressure*	Systolic	121.37 ± 25.83	126.62 ± 24.47	122.47 ± 25.52	0.430
	Diastolic	77.71 ± 17.15	82.48 ± 20.85	78.71 ± 17.98	
*Heart rate*	Beats/min	82.65 ± 16.54	89.86 ± 17.73	84.18 ± 16.97	0.489
*Body temperature*	Celsius	36.09 ± 3.33	36.65 ± 0.48	36.21 ± 2.98	0.196
*SpO2*	Percent	91.65 ± 5.1	92.57 ± 3.7	91.84 ± 4.84	0.102
*HF type*	Ischemic	36/45.6	7/33.3	43/43	0.683
	Non-ischemic	43/54.4	14/66.7	57/57	0.314
*AHF type*	ADHF	63/79.7	15/71.4	78/78	0.413
	DNHF	16/20.3	6/28.6	22/22	
*HF stage*	C	53/70.7	15/75	68/71.6	0.703
	D	22/29.3	5/25	27/28.4	
*NYHA*	III	36/45.6	9/42.9	45/45	0.992
	IV	19/24.1	5/23.8	24/24	
*Dyspnea*	-	72/91.1	16/76.2	88/88	0.061
*Orthopnea*	-	20/25.3	4/19	24/24	0.550
*Paroxysmal nocturnal dyspnea*	-	4/5.1	1/4.8	5/5	0.955
*Chest pain*	-	15/19	5/23.8	20/20	0.623
*Fatigue*	-	14/17.7	4/19	18/18	0.888
*Tachycardia*	-	12/15.2	2/9.5	14/14	0.506
*Edema*	-	50/50.6	5/23.8	45/45	**0.028**
*GI tract symptoms*	-	16/20.3	4/19	20/20	0.902
*Ascites*	-	10/12.7	0/0	10/10	0.086
*Elevated JVP*	-	11/13.9	1/4.8	12/12	0.251

**Table 4:** Medicines used by patients

Time	Medications	Group A (Frequency/Percentage)	Group B (Frequency/Percentage)	Total (Frequency/Percentage)	*P*-value
*Medications used before hospitalization*	Calcium channel blockers	3/4.4	2/11.1	5/5.8	0.280
	Furosemide	42/61.8	7/38.9	49/57	0.081
	ACE inhibitors	18/26.5	3/16.7	21/24.4	0.389
	Angiotensin receptor blockers	23/33.3	4/22.2	27/31	0.364
	Beta-blockers	40/58.8	4/22.2	44/51.2	**0.006**
	Spironolactone	24/35.3	3/16.7	27/31.4	0.130
	Digoxin	24/35.3	4/22.2	28/32.6	0.293
	Nitrate-based vasodilators	7/10.3	1/5.6	8/9.3	0.538
*Medications used during hospitalization*	ACE inhibitor	58/73.4	11/52.4	69/69	0.064
	Angiotensin receptor blockers	2/2.5	0/0	2/2	0.461
	Inotrope	24/30.8	3/14.3	27/27	0.132
	Nitrate-based vasodilators	9/11.4	0/0	9/9	0.105
	Spironolactone	58/73.4	17/22.7	75/75	0.478
	Beta-blockers	69/87.3	16/76.2	85/85	0.203
	Allopurinol	2/2.5	0/0	2/2	0.461
	Digoxin	30/38.5	2/6.3	32/32	**0.012**

**Table 5:** Laboratory findings

Variables	hyperuricemia Group (Mean ± SD)	normal uric acid Group (Mean ± SD)	Total (Mean ± SD)	*P*-value
*WBC*	9576 ± 4099	8871 ± 4002	9407 ± 4064	0.376
*Hemoglobin*	13.02 ± 2.85	12.97 ± 2.40	13.01 ± 2.74	0.869
*Hematocrit*	39.45 ± 7.70	39.63 ± 6.84	39.49 ± 7.47	0.784
*Platelet*	211,029 ± 83,503	228,990 ± 102,344	215,315 ± 88,073	0.484
*BUN*	37.17 ± 22.15	19.58 ± 8.27	33.48 ± 21.27	**< 0.001**
*Admission creatinine*	1.99 ± 1.16	1.31 ± 0.91	1.85 ± 1.15	**0.002**
*Discharged creatinine*	1.87 ± 0.21	1.05 ± 0.23	1.86 ± 1.74	**0.001**
*Admission eGFR*	44.03 ± 21.33	68.80 ± 39.06	47.84 ± 25.97	0.082
*Discharged eGFR*	44.11 ± 23.92	79.65 ± 47.84	49.68 ± 31.17	**0.033**
*eGFR changes*	10.49 ± 2.91	28.08 ± 16.21	2.83 ± 15.22	0.521
*Natrium [Na]*	137.22 ± 5.43	139.38 ± 3.44	137.68 ± 5.13	0.081
*Potassium [K]*	4.39 ± 0.75	4.24 ± 0.51	4.36 ± 0.71	0.709
*Uric acid*	9.43 ± 1.93	5.22 ± 1.31	8.55 ± 2.50	**< 0.001**
*Blood sugar*	179.90 ± 141.21	118.83 ± 95.52	167.89 ± 135	**0.022**
*LDL*	98.67 ± 72.49	74.53 ± 102.50	99.61 ± 72.35	0.636
*HDL*	35.52 ± 9.29	40 ± 11	36.64 ± 9.85	0.155
*Triglyceride [TG]*	109.19 ± 56.07	148.65 ± 136.41	85.24 ± 119.51	0.654
*AST*	89 ± 167.62	85.97 ± 179.55	86.61 ± 176.18	0.556
*ALT*	83.46 ± 189.25	81.83 ± 177.32	83.13 ± 185.91	0.620
*ALP*	246.91 ± 114.55	317.82 ± 377.49	260.77 ± 194.52	0.401
*Total bilirubin*	1.49 ± 1.18	0.95 ± 0.41	1.38 ± 1.09	0.087
*ESR*	42.47 ± 42.49	33.80 ± 20.64	4.40 ± 38.37	0.988
*CRP*	0.97 ± 1.31	1.11 ± 1.57	1 ± 1.37	0.903

**Table 6:** The cumulative dose and frequency of diuretics administered to patients during hospitalization

Variables	Route	hyperuricemia Group (Mean ± SD) (Frequency/Percentage)	Natural uric acid Group (Mean ± SD) (Frequency/Percentage)	Total (Mean ± SD) (Frequency/Percentage)	*P*-value
*Furosemide*	Total	645.52 ± 372.36	473.26 ± 391.45	609.95 ± 380.69	0.061
	Oral	136.44 ± 181.97	164.21 ± 203.59	142.17 ± 185.81	0.582
	IV	577.30 ± 400.63	391.16 ± 402.47	538.86 ± 405.92	**0.045**
*Thiazides*	Oral	4/5.1	0	4/4	0.576
*acetazolamide*	Oral	2/2.5	0	22/22	0.999

**Table 7:** Sub-gender analysis for cumulative dose and frequency of diuretics administered to patients during hospitalization

	Variables	Route	hyperuricemia Group (Mean ± SD) (Frequency/Percentage)	Natural uric acid Group (Mean ± SD) (Frequency/Percentage)	Total (Mean ± SD) (Frequency/Percentage)	*P*-value
Male	*Furosemide*	Oral	132.07 ± 180.30	129.09 ± 230.36	131.56 ± 187.77	0.685
		IV	570.83 ± 376.48	349.09 ± 226.86	532.71 ± 363.69	0.73
	*Thiazides acetazolamide*	Oral	570.83 ± 36.40	349.10 ± 226.87	5.38 ± 33.21	0.233
		Oral	36.363 ± 212.01	0.0 ± 0.0	30.30 ± 193.72	0.252
Female	*Furosemide*	Oral	139.09 ± 184.18	266.66 ± 146.78	166.42 ± 182.25	0.562
		IV	601.86 ± 492.99	373.33 ± 510.56	552.89 ± 496.41	0.881
	*Thiazides acetazolamide*	Oral	25.0 ± 85.97	0	19.35 ± 76.02	0.107
		Oral	0	0	0	-

**Table 8:** ECG findings

Variables	Type	hyperuricemia Group (Frequency/Percentage)	normal uric acid Group (Frequency/Percentage)	Total (Frequency/Percentage)	*P*-value
*Rhythm*	Normal sinus	51/64.6	15/71.4	66/66	0.801
	Ventricular tachycardia	1/1.3	0/0	1/1	
	Atrial fibrillation	25/31.6	5/23.8	30/30	
	Pacemaker	2/2.5	1/4.8	3/3	
*Wide QRS*	-	7/8.9	3/14.3	10/10	0.461
*LBBB*	-	14/17.7	1/4.8	15/15	0.139
*RBBB*	-	5/6.3	5/23.8	10/10	**0.018**
*Q wave*	-	14/17.7	1/4.8	15/15	0.139
*ST segment changes*	-	12/15.2	2/9.5	14/14	0.506

**Table 9:** Echocardiographic findings

Variables	Unit/Type	hyperuricemia Group (Mean ± SD) (Frequency/Percentage)	normal uric acid Group (Mean ± SD) (Frequency/Percentage)	Total (Mean ± SD) (Frequency/Percentage)	*P*-value
*LVEF*	%	19.71 ± 10.07	27.50 ± 11.79	21.34 ± 10.86	**0.011**
*LVED*	mm	57.4 ± 29.99	52.61 ± 10.18	56.36 ± 10.16	0.052
*LVES*	mm	48.50 ± 9.42	35.86 ± 5.58	45.22 ± 10.20	**0.001**
*LAVI* ^**1**^	mm	42.69 ± 12.43	26.33 ± 7.44	38.84 ± 13.32	**0.015**
*RAA*	mm	18.2 ± 13.12	11.48 ± 4.30	16.44 ± 4.52	**0.005**
*RVDD*	mm	37.68 ± 6.57	33.80 ± 5.94	36.82 ± 6.60	**0.049**
*TAPSE* ^**2**^	mm	16.41 ± 4.83	17.68 ± 2.58	16.71 ± 4.42	0.102
*Mitral regurgitation*	Absence	3/4.5	1/5.9	4/4.8	0.694
	Mild	13/19.4	5/29.4	18/21.4	
	Moderate	36/53.7	9/52.9	45/53.6	
	Severe	15/22.4	2/11.8	17/20.2	
*Tricuspid regurgitation*	Absence	5/7.6	0/0	5/6	0.123
	Mild	14/21.2	8/44.4	22/26.2	
	Moderate	43/65.2	8/44.4	51/60.7	
	Severe	4/6.1	2/11.1	6/7.1	

**Table 10:** Correlations between serum uric acid levels and lengths of admission with other variables

Variables	Serum uric acid level		Admission duration	
	r	*P*-value	r	*P*-value
*Serum uric acid level*	-	-	-0.122	0.230
*Admission duration*	-0.122	0.230	-	-
*Total furosemide*	0.291	**0.005**	0.612	**< 0.001**
*Oral furosemide*	-0.174	0.098	0.391	**< 0.001**
*IV furosemide*	0.313	**0.002**	0.485	**< 0.001**
*Thiazides*	0.154	0.063	0.117	0.178
*Acetazolamide*	0.084	0.313	0.124	0.153
*Furosemide before hospitalization*	0.282	**0.008**	-0.097	0.375
*Age*	0.169	0.093	0.025	0.804
*Weight*	0.3	**0.030**	-0.181	0.198
*LVEF*	-0.265	**0.014**	0.108	0.326
*LVED*	0.279	**0.014**	-0.295	**0.01**
*LVES*	0.578	**0.002**	-0.267	0.179
*LAVI*	0.458	**0.048**	-0.4	0.112
*RAA*	0.474	**0.040**	-0.554	**0.014**
*RVDD*	0.325	**0.007**	-0.18	0.145
*BUN*	0.371	**< 0.001**	0.113	0.265
*Creatinine*	0.323	**0.001**	0.068	0.504
*Blood sugar*	-0.006	0.963	0.023	0.858
*Discharged eGFR*	-0.17	0.234	-0.034	0.811

**Table 11:** Multivariate logistic regression was used to determine the relationship between the variables and the UA

Variables	Odds ratio	Confidence interval	*P*-value
*LVES*	0.876	0.490–1.565	0.654
*LVED*	1.087	0.552–2.142	0.809
*RVDD*	0.850	0.420–1.724	0.653
*BUN*	0.931	0.705–1.230	0.616
*RAA*	0.608	0.232–1.593	0.311

The other variables (Creatinine, LAVI, Weight, LVEF, furosemide) were removed from the model due to non-Linearity

**Table 12:** Univariate logistic regression to determine the association between the variables and in-hospital mortality

variables	Odds ratio	Confidence interval	*P*-value
*admission uric acid level*	0.615	0.408–0.929	**0.021**
*Age*	0.950	0.875–1.031	0.223
*Weight*	0.926	0.826–1.039	0.191
*Admission duration*	0.968	0.812–1.153	0.712
*Acetazolamide*	0.999	0.996–1.001	0.324
*Discharge eGFR*	1.003	0.955–1.053	0.910
*IV furosemide*	0.997	0.995–0.999	**0.013**
*Oral furosemide*	1.523	2.32	0.990
*Total furosemide*	0.997	0.995–1.00	**0.034**
*BUN*	0.960	0.927–0.994	**0.021**
*Creatinine*	0.492	0.274–0.882	**0.017**
*Blood sugar*	0.995	0.989–1.00	0.061
*LVED*	0.913	0.807–1.033	0.149
*LVEF*	0.986	0.910–1.068	0.723
*LVES*	0.738	0.468–1.162	0.190
*RVDD*	1.142	0.931–1.401	0.204

**Table 13:** Multivariate logistic regression was used to determine the relationship between the variables and the mortality rate independently

Variables	Odds ratio	Confidence interval	*P*-value
*Admission uric acid level*	0.771	0.490–1.215	0.262
*IV furosemide*	0.702	0.001 -	0.991
*Oral furosemide*	1.423	0.001 -	0.991
*BUN*	0.957	0.879–1.043	0.317
*Creatinine*	0.979	0.319–3.003	0.971

## Discussion

This study was designed to assess the relation between the serum UA levels and received dosage of diuretics during the hospitalization of HF. Based on our findings, serum UA levels are significantly associated with the received dosage of diuretics and also is an independent predictor of prognosis in HF patients.

Congestive heart failure (HF) is a major and growing public health problem. Right now more than 2 million people in united states of America have HF and this number is expected to increase in the upcoming decades [23]. HF can have a mortality rate up to 50% and About 35% of all patients with a diagnosis of HF are hospitalized every year [24]. hyperuricemia is very common in patients with HF and is associated with more advanced disease state. The source of UA is likely multifactorial and includes up-regulation of Xanthine oxidase (XO), a key enzyme in purine metabolism that derives reactive oxygen species responsible for deteriorative processes in HF like myocardial fibrosis, cardiac hypertrophy, left ventricular remodeling and impaired contractility. Impairment of endothelial cells by UA is another mechanism. up-regulation of catabolic pathways, insulin resistance, increased rates of cell and tissue wasting are other possible explanations. also, Sympathetic activation in HF could constrict renal glomerular arterioles leading to decrease of glomerular filtration rate and reduction of UA excretion and finally increase in UA levels. Hyperuricemia could activate the renin-angiotensin- aldosterone system and further ventricular remodeling in HF that eventually leading to poor prognosis [25–29]. In recent years, numerous epidemiological studies revealed the association between UA levels and various cardiovascular and cerebrovascular diseases including hypertension, coronary artery disease and HF. A recent systematic review and meta-analysis by Miao et al. concluded that high serum UA level independently could predict the risk of all-cause mortality, cardiovascular events and death in chronic heart failure [30]. Other studies claim that Serum UA level can be an independent prognostic factor in hospitalized HF patients. And also Hyperuricemia on admission is associated with the use of loop diuretics and the presence of chronic kidney disease [31]. In the study of maloberti et al. It was suggested that diuretic therapy could determine an increase in UA and also diuretic-related hyperuricemia is associated with all-cause mortality in cardiovascular patients [32]. the study of rebora et al. claims that admission UA levels can be a reliable predictor of worse ACS complications such as acute HF and cardiogenic, but they also claim that a worse presentation can be able to increase serum UA levels in ACS patients [33].In this study there was also a significant relationship between the admission uric UA level and the mortality rate in HF patients. Diuretics and their function in reducing body sodium and fluid are the cornerstone of HF therapy [34]. Most HF admissions are due to volume overload and treated with intravenous (IV) loop diuretics. However, there is currently no specific knowledge on adjustment of IV loop diuretic doses based on individual responses to initial diuretic. In fact, many patients are inadequately treated because of various diuretic dosing and responses [35]. Diuretic resistance is one of the most common challenges that physicians encountered during HF hospitalization and is related to worse prognosis. The furosemide dose before admission is an independent predictor of chronic drug resistance [36]. In a study by YAMAMOTO et al. HF patients with increased UA levels have received more dosage of loop diuretics. They also found that nearly 50% of subjects had an increase in UA during hospitalization for acute decompensated HF and that this increase was associated with long-term readmission but not with all-cause mortality. If the hypothesis that UA levels increase acutely during hospitalization due to secondary hemodynamic effects is true, we would expect to observe a decrease in the majority of patients from admission to discharge. However, other factors such as residual congestion, renal function impairment, and higher doses of loop diuretic could lead to an increase in UA levels during hospitalization [37]. Furthermore, Zhou et al. established that the alteration in GFR and the dosage of loop diuretics play a crucial role in the elevation of UA levels throughout the period of hospitalization [22]. Studies claim that diuretics can increase serum UA level by stimulating UA reabsorption in the proximal tubule, and diuretic induced elevations in serum UA are known to be dose dependent [38, 39]. In this study it was the same. As HF patients with a higher level of UA have received more dosage of diuretics during their hospitalization. This change in UA levels might be a consequence of treatment because diuretics may potentially increase serum UA levels by stimulating UA reabsorption in the proximal tubule. Another possible explanation for this matter is that UA has a relation with body mass index (BMI). And can be decreased by decongestion. Which is the main performance of diuretics [40, 41].

### Limitations

Our study had several limitations. First, data were generated from a single center. Second, due to the observational design of this study, it is impossible to prove causality and findings in our study are hypothesis generating. Third, is the limited sample size of the study. Fourth is the lack of long-term follow-up in the study. Finally, directionality of the relationship founded in our study could not be determined.

## Conclusion

This study found that a high level of UA in HF patients is significantly related to higher dosage of diuretics used for treatment of HF patients and in hospital mortality of them. Future prospective multicenter studies with larger sample size are needed to understand how UA affects the pathology of HF and whether interventions to hyperuricemia might benefit patients with HF.

### Electronic Supplementary Material

Below is the link to the electronic supplementary material.

Supplementary Material 1

## Data Availability

The datasets analyzed during the current study available from the corresponding author on reasonable request.
