# Supplementary material for: Could admission level of uric acid predict total diuretic dose in acute heart failure?
Source: BMC Cardiovasc Disord. 2024 Jan 3;24:30. doi: 10.1186/s12872-023-03687-w (PMC10765671; doi:10.1186/s12872-023-03687-w)
Supplement: Supplementary file 1 — Supplementary Material 1 [file 12872_2023_3687_MOESM1_ESM.docx]

| **Variables** | **hyperuricemia Group**  (Frequency/Percentage) | **normal uric acid Group**  (Frequency/Percentage) | **Total**  (Frequency/Percentage) | **P-value** |
| --- | --- | --- | --- | --- |
| **Failure to use medications** | 0/0 | 1/4.8 | 1/1 | 0.336 |
| **ACS** | 26/32.9 | 6/28.6 | 32/32 |  |
| **Anemia** | 1/1.3 | 1/4.8 | 2/2 |  |
| **Renal failure** | 7/8.9 | 1/4.8 | 8/8 |  |
| **Arrhythmia** | 20/25.3 | 4/19 | 24/24 |  |
| **Unknown causes** | 25/31.6 | 8/38.1 | 33/33 |  |

**Table 2 Causes of decompensated heart failure**

| **Time** | **Medications** | **Group A**  (Frequency/Percentage) | **Group B**  (Frequency/Percentage) | **Total**  (Frequency/Percentage) | **P-value** |
| --- | --- | --- | --- | --- | --- |
| **Medications used before hospitalization** | Calcium channel blockers | 3/4.4 | 2/11.1 | 5/5.8 | 0.280 |
|  | Furosemide | 42/61.8 | 7/38.9 | 49/57 | 0.081 |
|  | ACE inhibitors | 18/26.5 | 3/16.7 | 21/24.4 | 0.389 |
|  | Angiotensin receptor blockers | 23/33.3 | 4/22.2 | 27/31 | 0.364 |
|  | Beta-blockers | 40/58.8 | 4/22.2 | 44/51.2 | **0.006** |
|  | Spironolactone | 24/35.3 | 3/16.7 | 27/31.4 | 0.130 |
|  | Digoxin | 24/35.3 | 4/22.2 | 28/32.6 | 0.293 |
|  | Nitrate-based vasodilators | 7/10.3 | 1/5.6 | 8/9.3 | 0.538 |
| **Medications used during hospitalization** | ACE inhibitor | 58/73.4 | 11/52.4 | 69/69 | 0.064 |
|  | Angiotensin receptor blockers | 2/2.5 | 0/0 | 2/2 | 0.461 |
|  | Inotrope | 24/30.8 | 3/14.3 | 27/27 | 0.132 |
|  | Nitrate-based vasodilators | 9/11.4 | 0/0 | 9/9 | 0.105 |
|  | Spironolactone | 58/73.4 | 17/22.7 | 75/75 | 0.478 |
|  | Beta-blockers | 69/87.3 | 16/76.2 | 85/85 | 0.203 |
|  | Allopurinol | 2/2.5 | 0/0 | 2/2 | 0.461 |
|  | Digoxin | 30/38.5 | 2/6.3 | 32/32 | **0.012** |

**Table 4 Medicines used by patients**

| **Variables** | **Type** | **hyperuricemia Group**  (Frequency/Percentage) | **normal uric acid Group**  (Frequency/Percentage) | **Total**  (Frequency/Percentage) | **P-value** |
| --- | --- | --- | --- | --- | --- |
| **Rhythm** | Normal sinus | 51/64.6 | 15/71.4 | 66/66 | 0.801 |
|  | Ventricular tachycardia | 1/1.3 | 0/0 | 1/1 |  |
|  | Atrial fibrillation | 25/31.6 | 5/23.8 | 30/30 |  |
|  | Pacemaker | 2/2.5 | 1/4.8 | 3/3 |  |
| **Wide QRS** | - | 7/8.9 | 3/14.3 | 10/10 | 0.461 |
| **LBBB** | - | 14/17.7 | 1/4.8 | 15/15 | 0.139 |
| **RBBB** | - | 5/6.3 | 5/23.8 | 10/10 | **0.018** |
| **Q wave** | - | 14/17.7 | 1/4.8 | 15/15 | 0.139 |
| **ST segment changes** | - | 12/15.2 | 2/9.5 | 14/14 | 0.506 |

**Table 7 ECG findings**

| **Variables** | **Unit/Type** | **hyperuricemia Group**  (Mean ± SD)  (Frequency/Percentage) | **normal uric acid Group**  (Mean ± SD)  (Frequency/Percentage) | **Total**  (Mean ± SD)  (Frequency/Percentage) | **P-value** |
| --- | --- | --- | --- | --- | --- |
| **LVEF** | % | 19.71 ± 10.07 | 27.50 ± 11.79 | 21.34 ± 10.86 | **0.011** |
| **LVED** | mm | 57.4 ± 29.99 | 52.61 ± 10.18 | 56.36 ± 10.16 | 0.052 |
| **LVES** | mm | 48.50 ± 9.42 | 35.86 ± 5.58 | 45.22 ± 10.20 | **0.001** |
| **LAVI ^1^** | mm | 42.69 ± 12.43 | 26.33 ± 7.44 | 38.84 ± 13.32 | **0.015** |
| **RAA** | mm | 18.2 ± 13.12 | 11.48 ± 4.30 | 16.44 ± 4.52 | **0.005** |
| **RVDD** | mm | 37.68 ± 6.57 | 33.80 ± 5.94 | 36.82 ± 6.60 | **0.049** |
| **TAPSE ^2^** | mm | 16.41 ± 4.83 | 17.68 ± 2.58 | 16.71 ± 4.42 | 0.102 |
| **Mitral regurgitation** | Absence | 3/4.5 | 1/5.9 | 4/4.8 | 0.694 |
|  | Mild | 13/19.4 | 5/29.4 | 18/21.4 |  |
|  | Moderate | 36/53.7 | 9/52.9 | 45/53.6 |  |
|  | Severe | 15/22.4 | 2/11.8 | 17/20.2 |  |
| **Tricuspid regurgitation** | Absence | 5/7.6 | 0/0 | 5/6 | 0.123 |
|  | Mild | 14/21.2 | 8/44.4 | 22/26.2 |  |
|  | Moderate | 43/65.2 | 8/44.4 | 51/60.7 |  |
|  | Severe | 4/6.1 | 2/11.1 | 6/7.1 |  |

**Table 8 Echocardiographic findings**
